# Development of a Pediatric Blood Pressure Percentile Tool for Clinical Decision Support

**DOI:** 10.1001/jamanetworkopen.2022.36918

**Published:** 2022-10-17

**Authors:** Blake Martin, Peter E. DeWitt, David Albers, Tellen D. Bennett

**Affiliations:** 1 Section of Critical Care, Department of Pediatrics, University of Colorado School of Medicine, Aurora; 2 Children’s Hospital Colorado, Aurora; 3 Section of Informatics and Data Science, Department of Pediatrics, University of Colorado School of Medicine, Aurora

## Abstract

This diagnostic study assesses the ability of a pediatric blood pressure percentile tool to accelerate identification of children with hypertension and hypotension by clinicians and researchers.

## Introduction

Pediatric blood pressure percentiles (BPPs) are critical for diagnosis of several conditions, including hypertension and shock.^1,2,3^ However, many published BPPs only apply to children 1 year or older, only provide percentiles above the median,^3^ and often require patient stature (missing in up to 33% of children at pediatric ICU admission).^2^ Lack of readily accessible BPPs creates barriers to patient care and development of pediatric-specific clinical decision support tools. Therefore, we created the pedbp R package to compute BPP estimates by age, sex, and stature (known or unknown) supported by pediatric BP assessments from prior studies to accelerate identification of children with hypertension and hypotension.

## Methods

Between January 2019 and March 2022, we performed a literature search for pediatric (age 1 month to 18 years) population BP measurements published from January 1981 to January 2022. We extracted reported systolic BP (SBP) and diastolic BP (DBP) means (SDs) and/or percentiles. The BPPs by age, sex, and stature (if known) were estimated via gaussian distributions (eMethods in the Supplement). Using R, version 4.2.1 (R Project for Statistical Computing), we designed the new pedbp R package and an intuitive web-based version of the tool (eFigure in the Supplement) to facilitate conversion of BP measurements to BPPs using age, sex, and stature (if available) inputs. Both offer batch processing capability whereby more than 1 BP measurement can be converted to BPPs simultaneously by using input data provided in a vector (R package) or csv file (web tool) format. This report follows the STARD reporting guidelines for diagnostic studies. Development of the BPP tool was completed within a project approved by the University of Colorado’s institutional review board.

## Results

**Figure 1.**
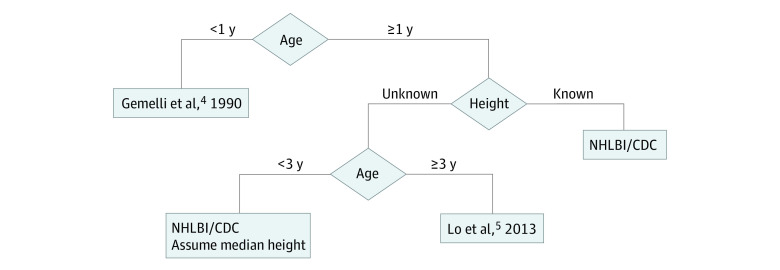
Flowchart for Data Source Determination Flowchart for determining the data source informing the mean and SDs for a gaussian distribution of blood pressures by age, sex, and stature (if known). CDC indicates Centers for Disease Control and Prevention; NHLBI, National Heart, Lung, and Blood Institute.

We identified reports^3,4,5^ that provided BP summary data for 32 227 children 1 to 17 years, 199 513 children 3 to 17 years, and 514 children 0 to 1 year of age. These data informed BP distribution design for each age, sex, and stature subgroup as follows (Figure 1): younger than 1 year, Gemelli et al;^4^ older than 1 year with known stature, National Heart, Lung, and Blood Institute (NHLBI) and Centers for Disease Control and Prevention (CDC) growth charts^3^; older than 3 years with unknown stature, Lo et al^5^; and age 1 to 3 years with unknown stature, NHLBI/CDC growth charts (assuming median stature for age and sex). The pedbp package (https://CRAN.Rproject.org/package=pedbp) and corresponding web-based tool (https://dewittpe.shinyapps.io/pedbp/) are publicly available.

An individual BPP can be obtained via a single R programming language function call. For example, a BP of 100/60 mm Hg in a 44-month-old boy with a stature of 105 cm is found via p_bp(q_sbp = 100, q_dbp = 60, age = 44, male = 1, stature = 105), which returns an SBP percentile of 70.86 and a DBP percentile of 84.86. Batch processing (generating BPPs for >1 observation) is performed by passing vectors of equal length for each argument (sbp, dbp, age, sex, and stature) to the p_bp function, which then returns SBP and DBP percentile vectors of equal length.

**Figure 2.**
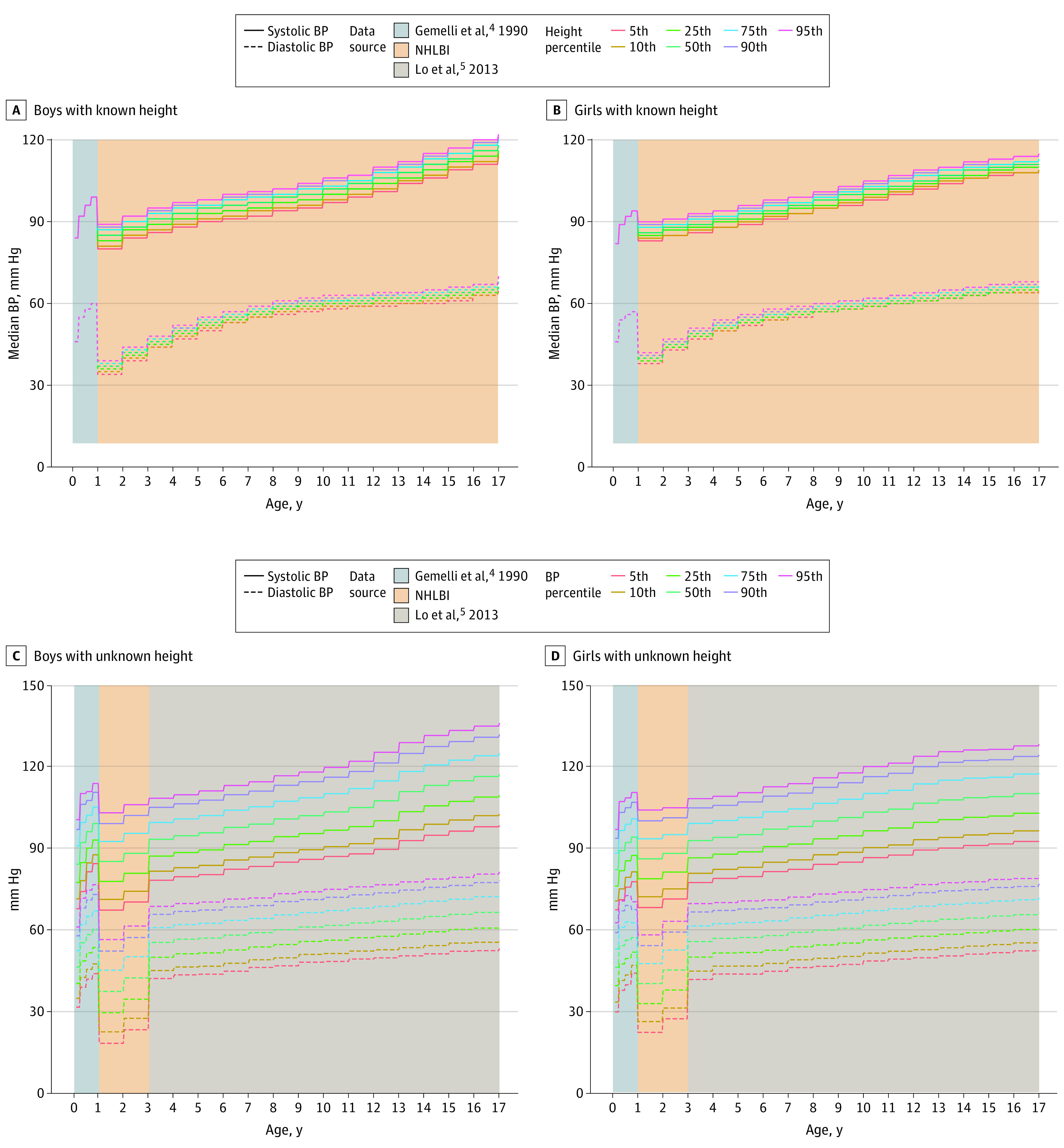
Systolic and Diastolic Blood Pressure (BP) Percentile Curves Graphs show the 50th-percentile systolic and diastolic BP curves at varying stature percentiles for boys (A) and girls (B), as well as various systolic and diastolic BP percentiles when a stature measurement is not available for boys (C) and girls (D). NHLBI indicates National Heart, Lung, and Blood Institute.

The BPPs for individuals can be determined using the web-based version by entering patient measurements into corresponding text boxes. Batch processing through the web-based application is accomplished by uploading a csv file with columns for each required patient input. The BPP results are then returned as a csv file. Additional examples, documentation, and features (including percentile charts [Figure 2]) are provided in the pedbp R package vignette.

## Discussion

The pedbp R package is a novel pediatric BPP reference tool capable of providing BPPs for all children regardless of stature measurement availability. This tool could aid pediatricians and researchers in interpreting vital signs, identifying children with hypertension or shock, and developing pediatric clinical decision support tools. A potential limitation is the assumption of gaussian BP distributions. Furthermore, because prematurity and several genetic and chronic medical conditions can affect patient stature and BP and critically ill children can have unique vital sign distributions,^6^ future pediatric BP studies should focus specifically on these subgroups to improve the source data informing the pedbp package. Future directions include creating BP distributions for these important pediatric subpopulations.

## Notes

Supplement.
eMethods.
**eFigure.** Screen Shot of the Web-Based Tool
