# Supplementary material for: Development of a Pediatric Blood Pressure Percentile Tool for Clinical Decision Support
Source: JAMA Netw Open. 2022 Oct 17;5(10):e2236918. doi: 10.1001/jamanetworkopen.2022.36918 (PMC9577675; doi:10.1001/jamanetworkopen.2022.36918)
Supplement: Supplement. — eMethods. eFigure. Screen Shot of the Web-Based Tool [file jamanetwopen-e2236918-s001.pdf]

Supplemental Online Content

Martin B, DeWitt PE, Albers D, Bennett TD. Development of a pediatric blood pressure percentile tool for clinical decision support. *JAMA Netw Open*. 2022;5(10):e2236918. doi:10.1001/jamanetworkopen.2022.36918

eMethods.
eFigure. Screen Shot of the Web-Based Tool

This supplemental material has been provided by the authors to give readers additional information about their work.

© 2022 Martin B et al. *JAMA Network Open.*

eMethods:

Creation of Blood Pressure Lookup Tables

After extracting systolic and diastolic BP summary statistics from the identified pediatric BP publications, we constructed a lookup table that lists the BP mean and standard deviation defining the Gaussian BP distribution for each age/sex/stature subgroup. For publications that reported certain BP percentiles (e.g. *75*th, 90th, 95th) but not the mean and standard deviation, we estimated mean and standard deviation parameters defining the Gaussian distribution such that the sum of squared differences between the Gaussian percentile and reported percentiles was minimized. Specifically, we optimized for the values of the mean (μ) and standard deviation (σ) such that for the provided quantiles q_i at the p_i percentiles and $X \sim N(\mu, \sigma)$,

$$\sum_i (\Pr(X \leq q_i) - p_i)^2$$

was minimized.

Conversion of Stature Measurements to Stature Percentiles

For conversion of patient BP measurements to BPPs when a stature measurement is known, conversion of the stature measurement to a stature percentile is necessary in order to utilize the NHLBI blood pressure charts. To determine the stature percentile for a given child (based on their age and sex), we utilized the LMS methods and data from the U.S. Centers for Disease Control and Prevention available at https://www.cdc.gov/growthcharts/percentile_data_files.htm.

© 2022 Martin B et al. *JAMA Network Open.*

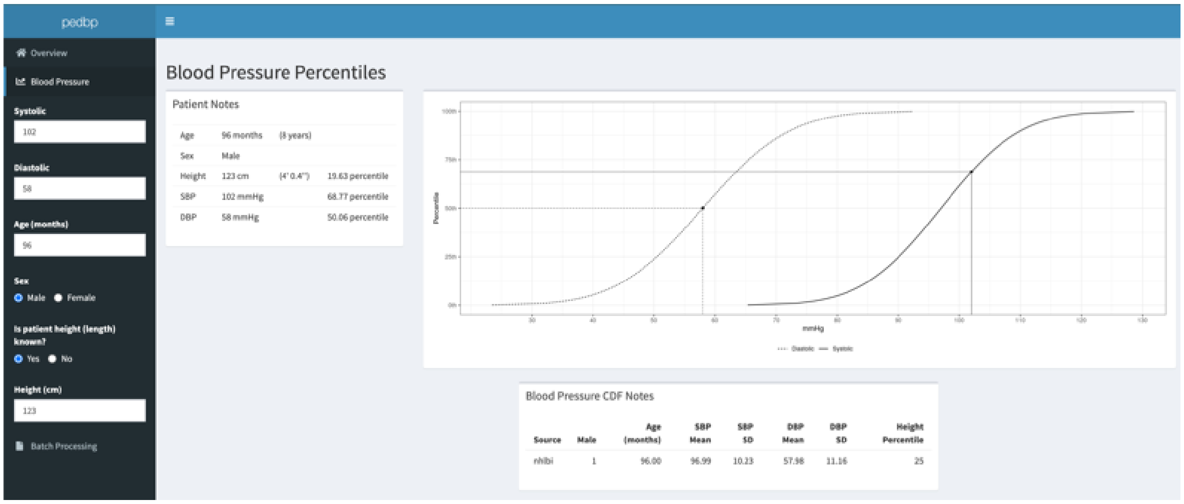

eFigure. Screen Shot of the Web-Based Tool

Screen shot of the freely available, interactive, web-based, blood pressure percentile tool available at the following url: https://dewittpe.shinyapps.io/pedbp/. This web version of the pedbp tool allows for conversion of blood pressure measurements to percentiles for individual and batch measurement processing.

© 2022 Martin B et al. *JAMA Network Open.*
